# Editorial: Metabolomics in crop research – current and emerging methodologies, volume III

**DOI:** 10.3389/fpls.2025.1627911

**Published:** 2025-06-02

**Authors:** Marta Sousa Silva, Carlos Cordeiro

**Affiliations:** FT-ICR and Structural Mass Spectrometry Laboratory, Biosystems and Integrative Sciences Institute (BioISI), Faculdade de Ciências, Universidade de Lisboa, Lisboa, Portugal

**Keywords:** metabolomics, agriculture, biomarkers, plant metabolism, defense mechanisms, plant-microbe interactions, quality control

The potential of metabolomics in plant and crop research was the driving force for the Research Topic “Metabolomics in Crop Research - Current and Emerging Methodologies” 8 years ago. Since then, this area has significantly evolved due to advances in analytical technologies, particularly mass spectrometry (MS), data processing, and integration with other “omics” fields. At the same time, artificial intelligence has improved the interpretation of complex metabolomic datasets, enabling researchers to link specific metabolic profiles with traits like stress tolerance, disease resistance, and nutritional content more effectively. Integration with genomics, transcriptomics, and phenomics became more straightforward, making metabolomics a central part of systems biology approaches to crop research.

The Research Topic “Metabolomics in Crop Research - Current and Emerging Methodologies” reached its third edition with 13 articles from 102 authors, highlighting different applications of metabolomics to plant metabolism and development, crop defense mechanisms, plant-microbe interactions and soil nutrient dynamics.

Understanding plant metabolism and development at the molecular level is crucial for improving crop quality, productivity, and resilience. Different analytical methodologies and “omics” strategies, particularly transcriptomics and metabolomics, are often combined to uncover the regulatory networks driving important phenotypic and metabolic outcomes in plants. In this Research Topic, Hu et al. explored the oil content of *Nicotiana tabacum* L. ‘Yunyan 87’ leaves as a crucial quality indicator, by combining a sensory evaluation with scanning electron microscopy (SEM) and GC×GC-TOF mass spectrometry. The high-oil tobacco leaves contained more metabolites than the low-oil content ones, with an increased concentration of oil- and aroma-related compounds. The oil of the palm *Elaeis guineensis* Jacq. was analyzed by Xu et al. to understand the regulatory mechanisms and metabolic pathways responsible for changes in oleic acid biosynthesis during fruit development. The metabolites from two oil palm varieties’ fruits (‘Seedless’ and ‘Tenera’) at three developmental stages were analyzed using a targeted metabolomics approach, by LC-MS^2^, combined with gene expression analysis of genes related to oleic acid metabolism. Significant differences were observed in both varieties and at the different fruit developmental stages and revealed the ley regulatory mechanisms underlying oleic acid biosynthesis. In another variety of the same crop plant, the green-fruited oil palm variety (‘O×G Amazon’), Zhang et al. analyzed the flavonoid content in fruits at different developmental stages. Flavonoids are important polyphenolic compounds with antioxidant and antimicrobial properties. The authors used a combined metabolomic (using an LC-TripleTOF) and transcriptomic approach to identify the compounds and key genes of flavonoid metabolism during fruit development. These studies paved the way for the development of metabolic engineering strategies for improving oleic acid and flavonoid content in oil palm plants. A similar research was performed by Wang et al. with different varieties of persimmon (*Diospyros kaki*) fruits, known by their rich flavonoid content. The authors analyzed the accumulation of flavonoids and its biosynthesis related genes’ expression in different developmental stages of two mature pollination-constant non-astringent varieties (‘Jiro’ and ‘Yohou’) and in two pollination-constant astringent ones (‘Zhongshi5’ and ‘Huojing’), using a targeted metabolomics and transcriptomic approach, relating them to the astringency and flavor traits of these persimmons’ fruits.

An integrative metabolomic and transcriptomic study by Wei et al. was also conducted in the flowers of *Chrysanthemum morifolium*, an important ornamental plant with culinary and medicinal value. Different colored petals (yellow, gold, and white) and as non-petal tissues were analyzed, revealing the key regulatory pathways responsible for color variation and providing a foundation for future quality control in breeding strategies. These *Chrysanthemum* plants are highly susceptible to pests, particularly to *Frankliniella occidentalis* (Pergande), a widely spread pest thrip affecting many ornamental plants and horticultural crops. Investigating the variety “Baltica White” from *C. morifolium*, Bierman et al. used metabolomics (1H NMR and headspace GC-MS) to study the effects of spraying of solutions containing sticky oil droplets, in reducing infection’s efficiency, in plant growth and in the leaf’s metabolome. The authors observed that these natural origin spraying solutions had a repellent or toxic effect for the thrips, leading to chemical changes in the plant leaves, thus playing a role in plant defense against these pests.

Cereals too are highly affected by pests, the wheat stem sawfly *Cephus cinctus* Norton being particularly harmful to wheat *Triticum aestivum* L. in North America. One of the most adopted strategies to prevent yield loss is the use of wheat varieties with solid stems, preventing the development of the larvae. Hager et al. studied the metabolome’s changes in response to infestation by the wheat stem sawfly in 2 varieties of oat (*Avena sativa* ‘Dane’ and ‘Otana’, resistant) and 4 varieties of spring wheat (*Triticum aestivum* ‘Choteau’, ‘Scholar’, ‘Conan’, and ‘Reeder’, with different levels of resistance), using an untargeted metabolomics approach by liquid chromatography-mass spectrometry (LC-MS). The analysis identified several compounds (carbohydrates, lipids and plant defense molecules) differentially expressed between samples and associated with the oat’s constitutive resistance to this sawfly, establishing the basis for the future development of new strategies to prevent wheat infection.

Besides the threats caused by insects, some crops must cope with invasive weeds, which compromise a significant percentage of the production yield. Rice (*Oryza sativa* L.) is one of these examples, largely affected by the invasive weed *Leptochloa chinensis*. Zhang et al. investigated the mutual inhibition of rice and this weed under mono- and co-culture conditions. A combined metabolomic (UHPLC-MS) and transcriptomic analysis revealed the metabolic and transcriptional regulatory networks behind the mutual suppression between rice and *L. chinensis*, with a more pronounced inhibition of root length in co-culture conditions. These results are highly relevant for developing new and more effective strategies for weed control and crop protection.

Crops are also affected by soil bacteria. These microorganisms can either cause damage, which is the case of the wilt causing agent *Ralstonia solanacearum*, or act as a community to provide natural defenses against pathogens and support healthy plant growth. In all these pathogenic or symbiotic networks, metabolites produced by plants and rhizosphere microorganisms play an important role. Wei et al. studied the metabolite composition of the rhizosphere of *Nicotiana tabacum* plants, cultivar ‘Yunyan 87’, grown in healthy and infected soils (by GC-TOF), the effect of bacterial communities, and the changes in soil properties. These results enlighten how rhizosphere bacteria confer resistance during the early stages of infection, thus contributing to a more sustainable control of soil pathogens. In another work, Wang et al. investigated the effect of the plant growth-promoting bacteria *Bacillus velezensis* in the growth of ramie (*Boehmeria nivea*), through the analysis of the soil microbial communities and the metabolic profile of the plant (by LC-MS). The yield and traits of ramie crop plants were improved after treatment with *B. velezensis*, revealing that these bacteria played an important role in regulating soil microbial structure and promoting plant metabolism, growth, and development, highlighting the importance of using these microorganisms as biological fertilizers and growth-promoting stimulants.

Plant growth is highly affected by the bioavailability of several nutrients, particularly nitrogen (N) and iron (Fe). Lodovici et al. studied the interplay between N and Fe nutritional pathways in tomato plants (*Solanum lycopersicum* L. cv ‘Marmande’) under both elements’ deficiency and after their reintroduction in the soil, by combining a physiological characterization with untargeted metabolomics (using UHPLC-QTOF) and gene expression analysis. The results revealed treatment-specific changes in tomato metabolic pathways, with changes in nitrogen-containing metabolites in leaves and roots and modulation of the phytohormone profile by the nitrogen source. This study contributes to understanding the interplay between nitrogen and iron nutritional pathways in crops.

Finaly, an important application of metabolomics in crop research is quality control and geographic origin. Sun et al. used an untargeted metabolomics approach (LC-MS/MS), combined with a targeted analysis for phenolic compounds (UHPLC-PDA), to analyze the Chinese dragon’s blood species *Dracaena cochinchinensis* and *Dracaena cambodiana* from distinct geographical origins. This study highlights the differences in the plant quality from different geographical origins and between the two species. Ji et al. presented a comprehensive review of the use of metabolomics in quality control of traditional Chinese medicinal plants. Their metabolism and metabolic profile were influenced by the plant’s geographical origin, developmental stage, harvesting cycle, and processing methods, thus strongly affecting the quality of different plant organs and their suitability for use in traditional Chinese medicine.

